# Digital 3D-Printed Surgical Guide for Removal of a Separated Instrument at the Maxillary Sinus Floor and Apical Microsurgery

**DOI:** 10.14740/jmc5361

**Published:** 2026-09-04

**Authors:** Bing Chun Li, Shu Ye, Cai Hong Lu, Guan Huang, Shuai Mei Xu

**Affiliations:** a Stomatological Hospital, School of Stomatology, Southern Medical University, Guangzhou, Guangdong Province, China; b Shenzhen UP3D Technology Co., Ltd., Shenzhen, Guangdong Province, China

**Keywords:** Chronic apical periodontitis, Instrument separation, Digital surgical guide, Endodontic microsurgery, Maxillary sinus

## Abstract

This case report describes a maxillary first molar with chronic apical periodontitis (CAP) and a separated endodontic instrument migrated to the maxillary sinus floor, which was successfully managed with digital guide-assisted endodontic microsurgery. A 20-year-old female patient presented with long-standing occlusal pain and discomfort in the left maxillary first molar. Clinical examination revealed a temporary restoration, tenderness to percussion, and no gingival swelling. During root canal treatment, the mesiobuccal (MB2) canal was found to be calcified and non-negotiable, and a small segment of a nickel-titanium instrument was separated and extruded beyond the apex. Periapical radiography and cone-beam computed tomography showed the separated nickel-titanium instrument exiting the MB root apex into the periapical region adjacent to the maxillary sinus floor. A diagnosis of CAP was established. Given the inaccessible MB2 canal and the separated instrument located outside the apex, conventional endodontic retreatment was not feasible. Accordingly, a digital surgical guide was designed to precisely localize the separated fragment, followed by apical microsurgery including apicoectomy, cyst debridement, instrument retrieval, retrograde preparation, and iRoot BP filling. At the 2-year follow-up, the periapical radiolucency had nearly disappeared, maxillary sinus floor mucosal thickening was significantly relieved, and the affected tooth maintained normal function after crown restoration. This is the first reported case, to our knowledge, in which a digital 3D-printed surgical guide-assisted microsurgery successfully removed a separated instrument beneath the maxillary sinus floor and preserved the tooth without sinus complications.

## Introduction

Chronic apical periodontitis (CAP) is a common oral inflammatory disease in endodontics, primarily attributed to persistent intraradicular microbial infection [1, 2]. Root canal treatment (RCT) is the conventional therapy for CAP; however, a considerable proportion of cases remain uncured after initial RCT, failure occurs in 7–18% of initial RCT [3, 4]. The common causes of failure include complex root canal anatomy, missed or calcified canals, persistent intracanal infection, separated instruments, and vertical root fractures [3, 5].

To address these failures, apical surgery has emerged as the primary treatment modality for refractory cases. With the advancement of surgical instruments, the incorporation of the dental operating microscope has significantly improved the success rate of apical surgery [6, 7]. Microscopic apical surgery allows direct removal of periapical lesions, resection of the susceptible apical 3 mm, thorough root-end preparation, and subsequent root-end filling with biocompatible materials [8, 9].

Nevertheless, even with microscopic surgery, performing apical surgery freehand remains challenging, particularly for less experienced clinicians or for posterior teeth with complex anatomy [10, 11]. The application of digital guides enables precise preoperative planning and accurate intraoperative navigation, thereby further enhancing the success rate of treatment for complex cases [12].

Herein, we report a case of CAP with a separated instrument in the maxillary left first molar, which was treated using digital guide-assisted microscopic apical surgery and achieved a favorable clinical outcome, providing a reference for the management of similar cases.

## Case Report

### Investigations

A 20-year-old female patient had been experiencing pain and discomfort in the left posterior maxillary tooth for several years. She denied any history of systemic diseases or drug allergies. The left posterior maxillary tooth had undergone resin composite restoration at a local dental clinic 7 years prior, with no history of previous root canal treatment. The patient gradually developed occlusal pain and discomfort, with no other associated symptoms, which had worsened over the previous 6 months. Following examination at our hospital, a diagnosis of CAP of the left maxillary first molar was made, and root canal treatment was recommended. During the root canal procedure on the left maxillary first molar, a nickel-titanium instrument approximately 2 mm in length separated and was extruded through the mesiobuccal (MB) apical foramen into the periapical region. The separated instrument was a Waveone Gold NiTi file (Dentsply Sirona, Ballaigues, Switzerland), with a diameter of 0.25 mm at the tip and a fractured segment length of approximately 2 mm. The fragment was located outside the apical foramen, having been extruded beyond the apex into the periapical tissue. Meanwhile, during retreatment, the MB2 canal was found to be calcified and inaccessible. The MB, distobuccal (DB), and palatal (P) canals were subsequently obturated, and further management was planned for the left maxillary first molar.

### Diagnosis

**Figure 1:**
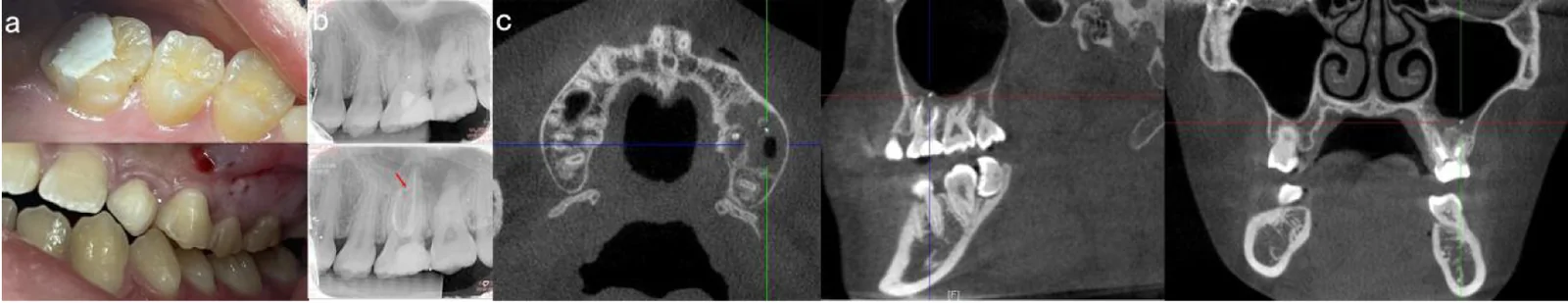
Diagnosis. (a) Preoperative clinical images: occlusal and buccal views, showing white temporary filling material on the occlusal surface. (b) Periapical radiographs of tooth #26 taken before and after root canal treatment (prior to apical surgery). The red arrow indicates the separated endodontic instrument. (c) Preoperative left maxillary CBCT scan, sagittal and axial slices. CBCT: cone-beam computed tomography.

As shown in Figure 1, clinical examination revealed a white temporary restoration on the left maxillary first molar (tooth #26). The tooth exhibited tenderness to percussion, normal physiological mobility, and no obvious gingival redness, swelling, or suppuration. Periapical radiography and cone-beam computed tomography (CBCT) showed radiopaque filling material in the crown and pulp chamber of tooth #26. The radiopaque fillings in the MB, DB, and P canals extended to the apices, whereas the MB2 canal was indistinct. The periodontal ligament space was slightly widened, and an ill-defined radiolucent shadow was observed in the MB periapical region. Bone destruction of the left maxillary sinus floor and thickening of the sinus mucosa were also noted, with a radiopaque separated instrument visible between the MB root of tooth #26 and the maxillary sinus floor. The left maxillary first molar was diagnosed with CAP.

### Treatment

In this case, the separated instrument had migrated outside the apex and was in close anatomical proximity to the maxillary sinus. Removing the fractured instrument via an intracanal approach was extremely challenging. Furthermore, the MB2 canal was calcified and non-negotiable, making it impossible to achieve a tight apical seal in the MB root, thereby hindering the resolution of periapical inflammation. Therefore, apical surgery was considered the optimal option to address both intractable problems simultaneously.

Nevertheless, apical surgery is associated with relatively extensive surgical trauma, intraoperative complications such as maxillary sinus perforation, and postoperative adverse reactions including pain and swelling. After detailed discussion and informed consent, the patient ultimately opted for surgical treatment.

Imaging examinations revealed that the separated instrument was located between the MB apical region of tooth #26 and the maxillary sinus floor. Radiographic bone loss at the maxillary sinus floor was noted, with only thickened sinus mucosa observed, indicating a high risk of sinus floor perforation and bone damage during surgical instrument removal. Accordingly, a digital surgical guide was prepared for intraoperative use to assist in precise localization.

The microsurgery approach advocated by Kim & Kratchman [13] was performed, by using the operative microscope magnification, ultrasonic tips, and bioceramic materials. The next clinical sequence was followed:

#### 1. Design and try-in of the digital surgical guide

**Figure 2:**
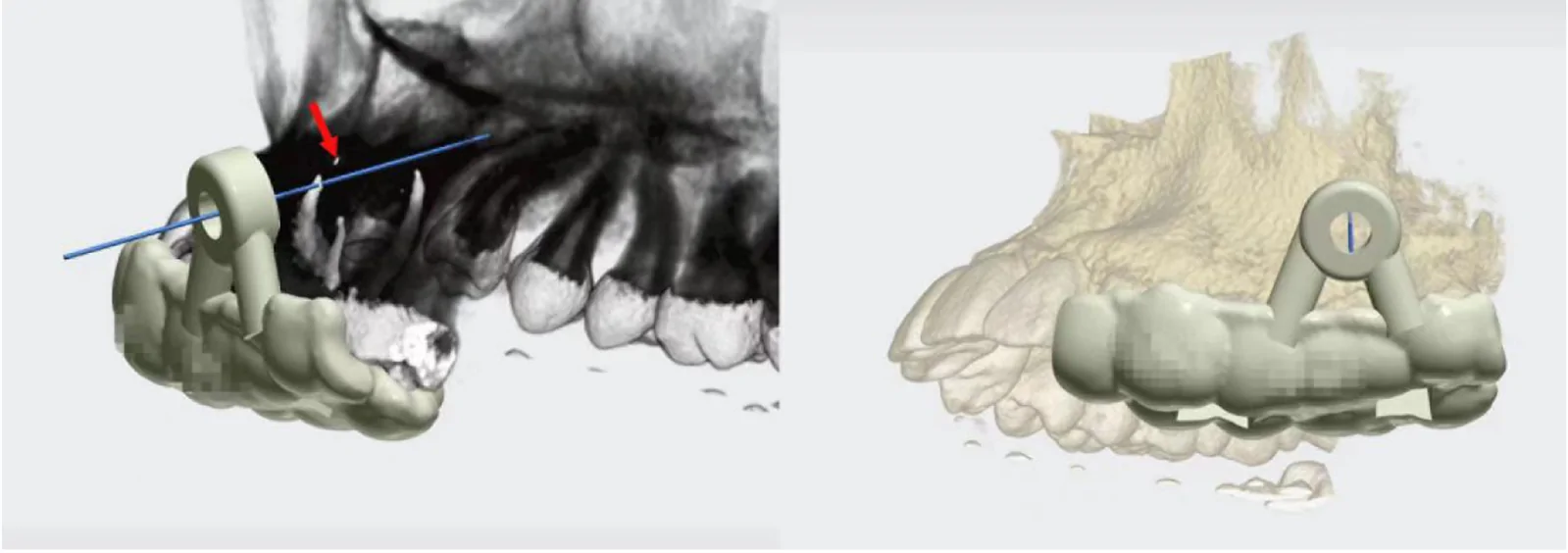
Digital surgical guide designed with 3Shape Unite software; the red arrow points to the separated endodontic instrument.

Preoperative digital design was performed using 3Shape Unite software. Based on CBCT data, the affected tooth and surrounding anatomical structures were reconstructed to create a personalized surgical guide. The apical incision site, osteotomy range, and removal pathway for the separated instrument were precisely planned to avoid injury to the maxillary sinus and adjacent anatomical structures. The surgical guide was fabricated using liquid crystal display (LCD) three-dimensional (3D) printing technology with medical-grade photosensitive resin (UP3D, CN), with a layer thickness of 0.1 mm and an overall printing accuracy of ± 0.2 mm. The 3D-printed surgical guide was then seated intraorally to verify its fit and positioning accuracy, thereby confirming the safety and feasibility of the surgical approach (Fig. 2).

#### 2. Anesthesia and flap elevation

**Figure 3:**
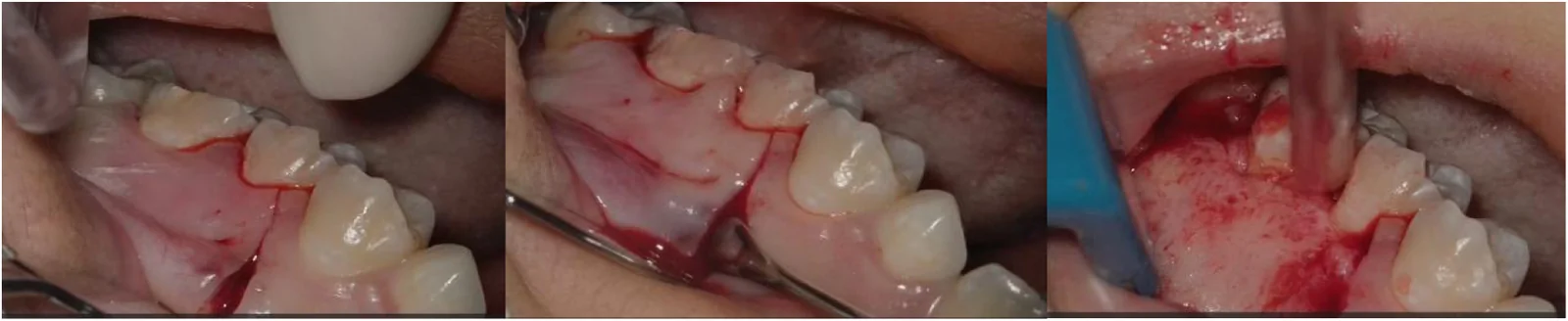
Angular flap (#25–#27 buccal, intrasulcular), full-thickness vertical/horizontal incisions, flap elevation, bone exposure.

Local anesthesia was administered to achieve intraoperative pain relief and localized hemostasis. An angular flap incision was made on the buccal side of teeth #25–#27, incorporating an intrasulcular incision, followed by full-thickness vertical and horizontal incisions and flap elevation to fully expose the bony surface of the surgical area (Fig. 3).

#### 3. Osteotomy and apical exposure

**Figure 4:**
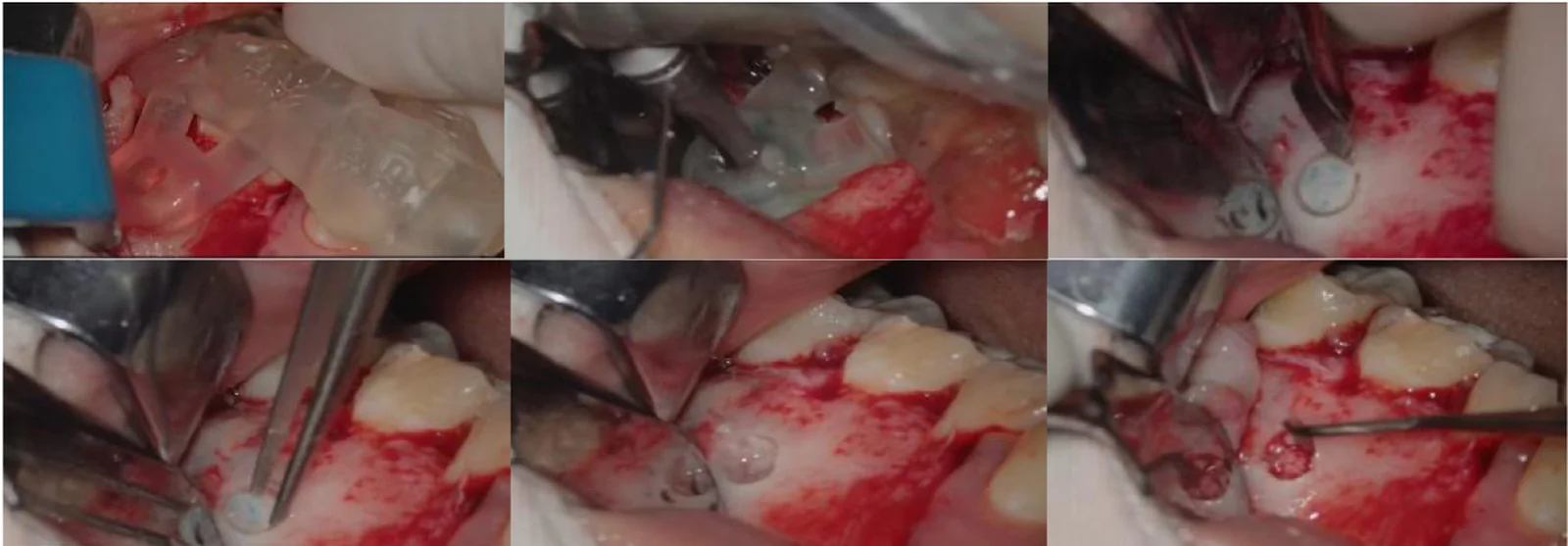
Digital surgical guide seated, trephine windowing, buccal bone plate removal, and apical localization.

The surgical guide was repositioned and seated. Under the guidance of the 3D-printed guide, a round bur was used for osteotomy and bone windowing. The MB apical region of tooth #26 was precisely located, and the round bony plate on the buccal side was removed to fully expose the apex of tooth #26 (Fig. 4).

#### 4. Removal of the separated instrument and apical management

**Figure 5:**
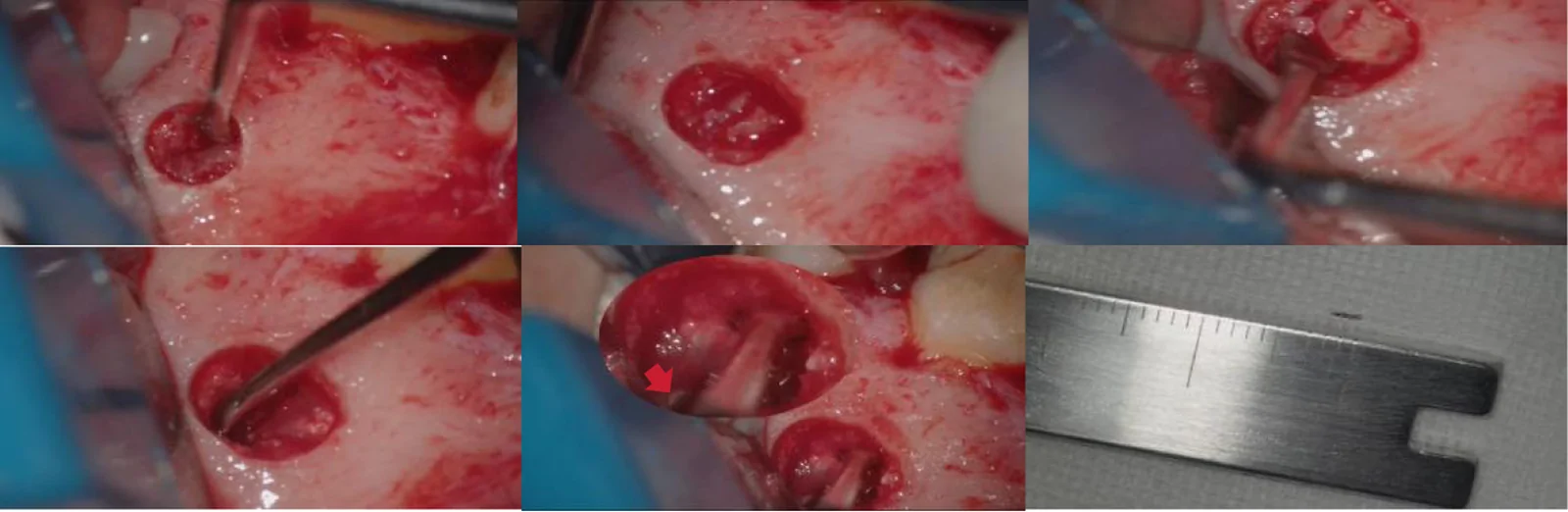
Apicoectomy of tooth #26, debridement of the periapical cyst, and removal of the separated instrument, the red arrow points to the separated endodontic instrument.

Apicoectomy was performed on tooth #26. Cyst-like granulation tissue in the apical region was thoroughly debrided, and the fractured nickel-titanium instrument inside the root canal was carefully extracted (Fig. 5).

#### 5. Retrograde preparation

**Figure 6:**
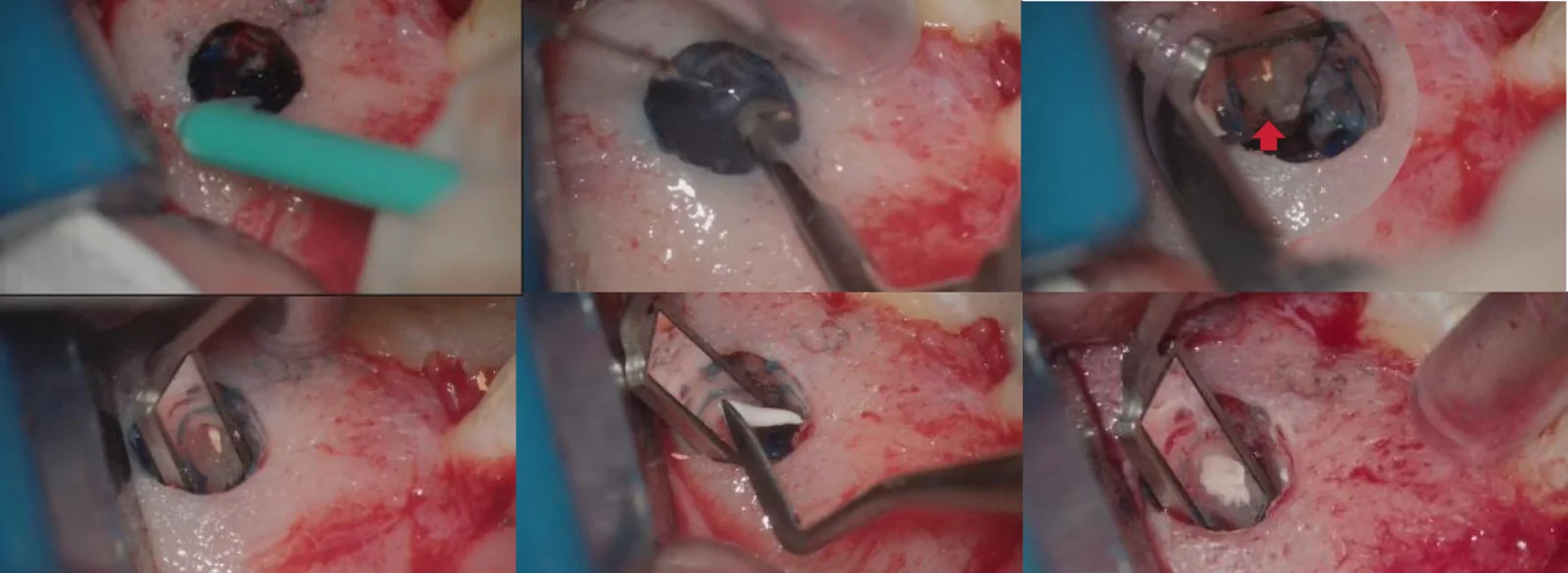
Methylene blue staining, retrograde preparation, and retrograde filling. Developmental malformation of the mesiobuccal root (red arrow).

A 3-mm retrograde preparation was completed for the MB root canal of tooth #26 to remove infected dentin. A developmental malformation was noted in the MB root intraoperatively and was carefully preserved during the procedure (Fig. 6).

#### 6. Retrograde filling

iRoot BP, a biocompatible material, was applied for retrograde filling of the MB root canal of tooth #26 (Fig. 6).

#### 7. Cleaning of the surgical site

Visual and radiographic examinations of the surgical area were performed. The osteotomy site was irrigated with normal saline to remove residual biomaterials and hemostatic agents.

#### 8. Flap repositioning and suturing

The flap was repositioned and sutured using 6-0 monofilament nylon suture (Ethicon, Johnson & Johnson Medical Devices & Diagnostics Group, Somerville, NJ, USA).

#### 9. Following the surgical procedure, a postoperative radiograph was taken

Postoperative care instructions for this type of surgery were provided, including flushing of the area with 0.12% chlorhexidine twice a day for 7 days, and the administration of 600 mg ibuprofen for 72 h.

### Follow-up and outcomes

**Figure 7:**
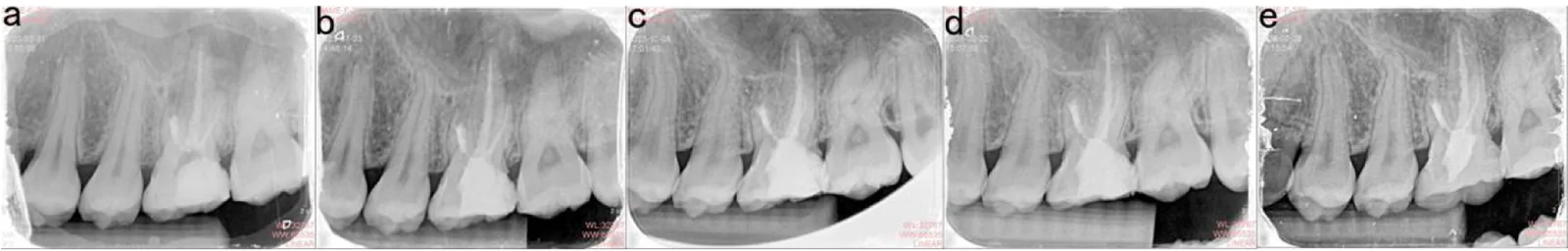
Postoperative periapical radiographs. (a) Immediate postoperative; (b) 4-month follow-up; (c) 9-month follow-up; (d) 1-year follow-up; (e) 2-year follow-up.

**Figure 8:**
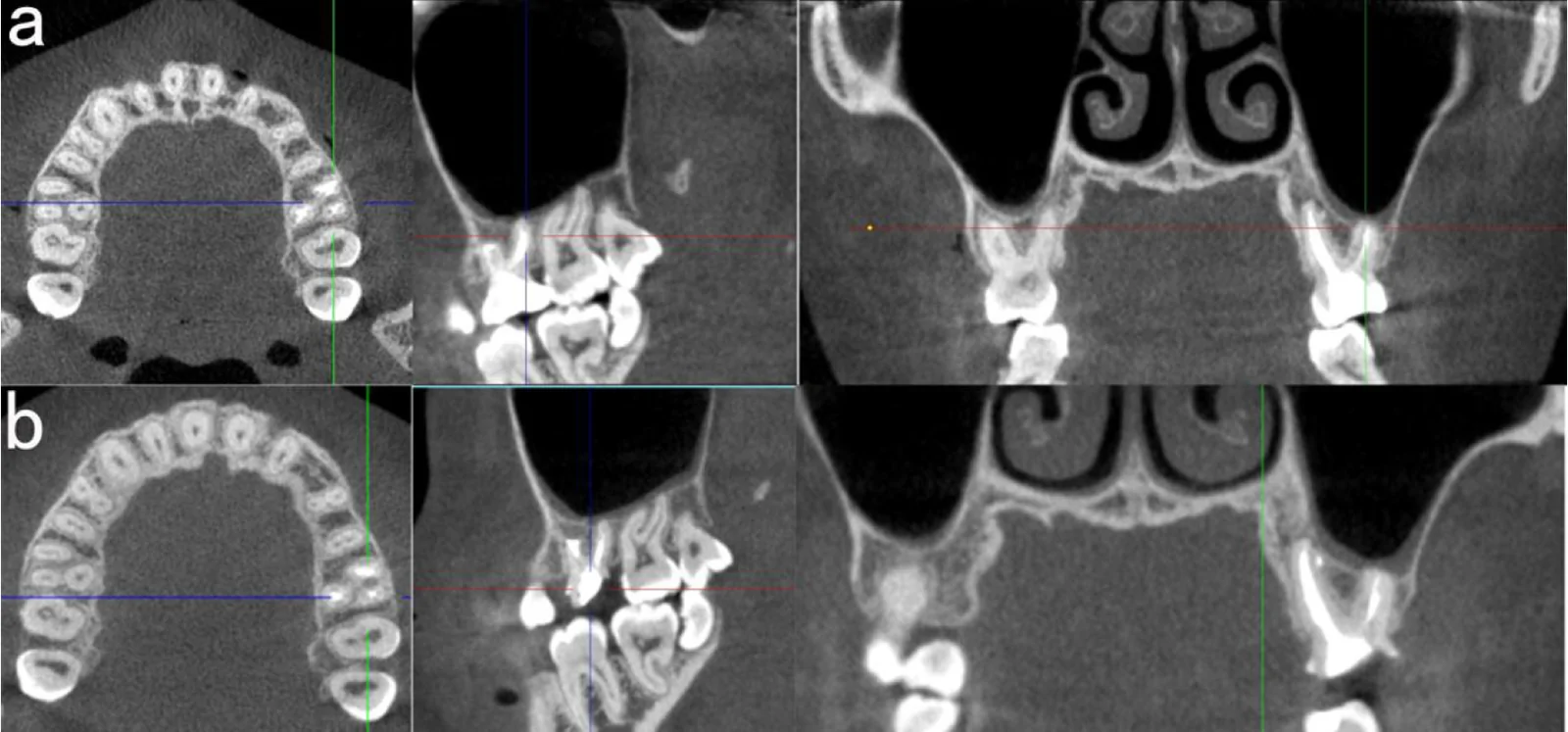
Postoperative CBCT. (a) 1-year follow-up; (b) 2-year follow-up. CBCT: cone-beam computed tomography.

**Figure 9:**
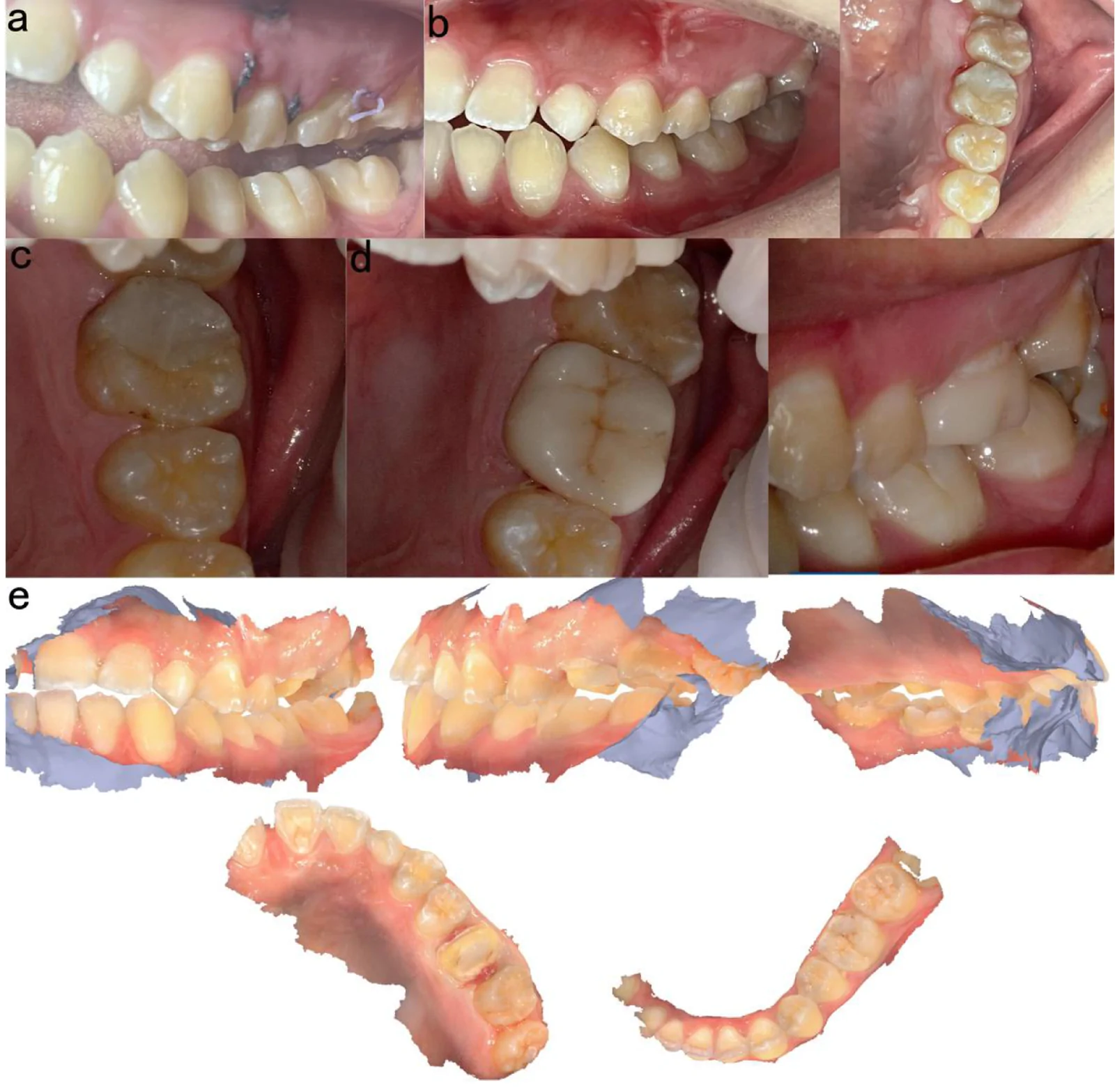
Intraoral photographs. (a) Before suture removal (10 days postoperatively); (b) 1-year follow-up; (c) 2-year follow-up; (d) Intraoral view after crown restoration (2 years); (e) Intraoral scan of the crown restoration.

Sutures were removed at 10 days postoperatively. The patient reported an uneventful recovery without obvious pain, swelling or maxillary sinus-related discomfort. At the 4-month postoperative follow-up (Fig. 7b), the periapical radiolucency had decreased by approximately 50% in diameter compared with preoperative measurements. At the 9-month follow-up (Fig. 7c), periapical radiography showed continued reduction of the radiolucent area. At the 1-year (Figs. 7d and 8a) and 2-year follow-ups (Figs. 7e and 8b), the radiolucency had nearly disappeared, with the defect area reduced by over 90%. Meanwhile, the thickening of the maxillary sinus floor mucosa was greatly relieved. The affected tooth maintained normal masticatory function without any discomfort. Eventually, crown restoration was performed on tooth #26 (Fig. 9).

## Discussion

The maxillary first molar is frequently subjected to root canal treatment and also exhibits the highest failure rates among all teeth. This is often attributed to the presence of a second canal in the MB2 that the operator fails to detect, debride, or obturate [14]. In approximately two-thirds of maxillary first molars, an MB2 canal is present [15]. Moreover, due to the close anatomical relationship between the MB root and the maxillary sinus, periapical inflammation in this tooth tends to result in persistent maxillary sinusitis.

Instrument separation is a relatively common complication during endodontic procedures, with an estimated incidence ranging from 1.83% to 8.2% [16]. The causes are multifactorial, including anatomical factors (e.g., canal curvature, calcification, obstruction, narrowing, and other complex anatomical variations), instrument-related factors, and operator-related factors. The likelihood of successful retrieval of a separated instrument is higher when the fragment is located in the middle or coronal third of the canal, but becomes significantly lower when it is confined to the apical third [17]. In the present case, the separated nickel-titanium instrument was extruded beyond the apex and had migrated to the maxillary sinus floor, making intracanal retrieval extremely unlikely.

Based on these challenges, the core difficulties encountered in this case were: (1) calcification and inaccessibility of the MB2 canal of tooth #26, which precluded complete infection elimination via conventional root canal retreatment; (2) localization of the separated instrument in the periapical region immediately adjacent to the maxillary sinus floor, CBCT revealed bony destruction of the maxillary sinus wall, with only a thin residual layer of mucosa remaining, rendering traditional blind retrieval techniques prone to complications such as sinus perforation; and (3) the presence of a developmental malformation in the MB root, further increasing anatomical complexity.

Endodontic microsurgery performed under high-magnification microscopy allows clear differentiation of periapical tissues, the separated fragment, and relevant anatomical structures, facilitating thorough debridement of granulation tissue and infected dentin [13]. When combined with a biocompatible retrograde filling material, it effectively seals the root apex, promotes bone healing, and provides a reliable means of preserving the affected tooth, with success rates between 88.9% and 100% [18, 19]. Despite the high reported success rate, the surgical procedure remains technically demanding [20]. This is particularly true for posterior teeth, where a smaller osteotomy makes intraoperative localization of the root apex more challenging [21, 22]. Therefore, accurate localization and precise, measured root-end resection are essential to minimize damage to adjacent anatomical structures, reduce postoperative pain, and promote healing of periapical lesions [23, 24].

To address these issues, a digital surgical guide was employed. The combination of a 3D-printed surgical guide and a trephine bur enables conservative osteotomy and precise root-end resection [25, 26]. This technology, based on preoperative CBCT-derived precise modeling, enables visualized planning of the surgical approach. Intraoperatively, it allows accurate guidance for ostectomy, apical localization, and retrieval of the separated instrument, thereby significantly reducing surgical risks, avoiding injury to the maxillary sinus and adjacent roots, shortening operative time, and enhancing treatment precision. In some case reports, the separated instrument was retrieved through free-hand microscopic endodontic surgery. Clinically, few case reports have described the removal of separated instruments located in the middle-apical segment of the root canal or extruded beyond the apical foramen. Mantri and Liu & Shen have reported successful retrieval using freehand endodontic microsurgery [27, 28]. Sudha et al applied 3D-printed surgical guides to precisely localize the osteotomy site for retrieving separated instruments in the apical root canal [29]. Yang & Chen used a 3D printing guide and trephine bur to retrieve a separated instrument beyond the apical foramen of the left maxillary central incisor [30]. Kaddoura et al reported the use of a 3D-printed surgical guide and a trephine bur for precise endodontic microsurgery in a mandibular first molar with a separated instrument and periapical lesions [31]. There are no reported cases of removing separated instruments located beneath the mucosa of the maxillary sinus floor.

This is the first clinical case in which a separated instrument located at the maxillary sinus floor was successfully removed using a combination of a digital 3D-printed surgical guide and endodontic microsurgery. Additionally, the periapical inflammation of the maxillary first molar was completely resolved, achieving favorable clinical outcomes. When surgical procedures involve vital anatomical structures such as the maxillary sinus, digital surgical guides can assist in accurately localizing separated instruments, reducing surgical invasiveness, lowering technical difficulty, and minimizing the incidence of intraoperative complications. In this case, a trephine bur with a 4-mm diameter was used for osteotomy, which ensured smooth surgical access and complete root-end resection while minimizing bone removal to the greatest extent.

As a single-center case report, this study has a limited sample size, and long-term outcomes require further follow-up. Future large-sample cohort studies are warranted to validate the clinical value of digital guide-assisted microsurgical apical surgery in complex periapical lesions and to optimize surgical protocols and material selection.

### Conclusion

In such challenging clinical cases, the use of positioning tools such as surgical guides helps reduce surgical difficulty, limit cortical bone destruction, and improve the success rate.

### Learning points

This case illustrates that a digital 3D-printed surgical guide, when combined with endodontic microsurgery, offers a precise and safe approach to retrieve a separated instrument that has extruded beyond the apex and lies adjacent to the maxillary sinus floor. Guided osteotomy not only minimizes cortical bone removal and reduces the risk of sinus perforation, but also facilitates simultaneous management of a calcified MB2 canal through retrograde preparation and bioceramic filling. Long-term follow-up with CBCT is indispensable to confirm periapical bone healing and resolution of sinus mucosal thickening, which are critical indicators of success in such anatomically challenging cases.

## Data Availability

The authors declare that data supporting the findings of this study are available within the article.
